# Surface Plasmon Resonance Reveals a Different Pattern of Proinsulin Autoantibodies Concentration and Affinity in Diabetic Patients

**DOI:** 10.1371/journal.pone.0033574

**Published:** 2012-03-19

**Authors:** Aldana Trabucchi, Luciano L. Guerra, Natalia I. Faccinetti, Rubén F. Iacono, Edgardo Poskus, Silvina N. Valdez

**Affiliations:** School of Pharmacy and Biochemistry, University of Buenos Aires (UBA), and Humoral Immunity Institute Prof. Ricardo A. Margni (IDEHU), National Research Council (CONICET)-UBA, Buenos Aires, Argentina; Catholic University Medical School, Italy

## Abstract

Type 1 diabetes mellitus (DM) is characterized by autoimmune aggression against pancreatic beta cells resulting in absolute deficiency of insulin secretion. The first detectable sign of emerging autoimmunity during the preclinical asymptomatic period is the appearance of diabetes-related autoantibodies. In children at risk for type 1 DM, high-affinity Insulin autoantibodies reactive to proinsulin, are associated with diabetes risk. Autoantibodies are usually measured by radioligand binding assay (RBA) that provides quasi-quantitative values reflecting potency (product between concentration and affinity) of specific autoantibodies. Aiming to improve the characterization of the specific humoral immune response, we selected surface plasmon resonance (SPR) as an alternative method to measure proinsulin autoantibodies (PAA). This novel technology has allowed real time detection of antibodies interaction and kinetic analysis. Herein, we have employed SPR to characterize the PAA present in sera from 28 childhood-onset (mean age 8.31±4.20) and 23 adult-onset diabetic patients (≥65 years old, BMI<30) in terms of concentration and affinity. When evaluating comparatively samples from both groups, childhood-onset diabetic patients presented lower PAA concentrations and higher affinities (median 67.12×10^−9^ M and 3.50×10^7^ M^−1^, respectively) than the adults (median 167.4×10^−9^ M and 0.84×10^7^ M^−1^, respectively). These results are consistent with those from the reference method RBA (Standard Deviation score median 9.49 for childhood-onset group and 5.04 for adult-onset group) where the binding can be directly related to the intrinsic affinity of the antibody, suggesting that there is a different etiopathogenic pathway between both types of clinical presentation of the disease. This technology has shown to be a useful tool for the characterization of PAAs parameters as an alternative to radioimmunoassay, with high versatility and reproducibility associated to low occupational and environmental risk. However, this technology is not eligible for routine marker screening, but this is a powerful technique for a fine description of the thermodynamic parameters of antigen-antibody interaction.

## Introduction

Type 1 diabetes mellitus (DM) is characterized by autoimmune aggression against pancreatic beta cells resulting in absolute deficiency of insulin secretion, and by its association with certain high-susceptibility HLA alleles. The first detectable sign of emerging beta cell autoimmunity during the preclinical asymptomatic period is the appearance of diabetes-related autoantibodies. On the other hand, type 2 DM occurs later in life and is characterized by insulin resistance and/or inadequate compensatory insulin secretion. Some adult patients with a phenotype of type 2 DM elicit antibodies directed against islet beta cell antigens. These patients, who slowly progress to insulin deficiency, are considered to have slow-onset or latent autoimmune diabetes (LADA) [1].

During the natural history of childhood diabetes, insulin and proinsulin autoantibodies (IAA/PAA) are often the first markers detected early in infancy [2], [3], [4], [5]. Many, but not all, IAA-positive children also develop autoantibodies to other beta cell antigens [3], [4], [6], [7]. Children who develop these additional antibodies usually progress to clinical type 1 DM, whereas those who remain positive only for IAA rarely develop the disease [3]. Therefore, development of multiple islet autoantibodies is an important feature in the pathogenesis of DM.

Achenbach et al. described in children at risk for type 1 DM, that high affinity IAA were associated with HLA DRB1*04, young age of IAA appearance, and subsequent progression to multiple islet autoantibodies or type 1 DM. In addition, high affinity IAA were reactive against proinsulin indicating that type 1 DM is associated with sustained early exposure to proinsulin in the context of HLA DR4, and showing that high-affinity proinsulin-reactive IAA identify children with the highest diabetes risk [8].

Conventionally, IAA/PAA are measured by radioligand binding assay (RBA), which requires radiolabeled antigen and reaction conditions to reach binding equilibrium (at least 4 days of incubation) to achieve higher signals. The quasi-quantitative values yielded by this method [9] reflect the influence of antibody concentration and affinity [10].

Aiming to characterize the specific humoral immune response against proinsulin, we selected surface plasmon resonance (SPR) as an alternative method to measure PAA through the assessment of antigen-antibody interaction parameters. It is important to note that in SPR assays such interaction can be expressed in terms of kinetic association (k_1_) and dissociation (k_−1_) rates besides the equilibrium affinity constant (K_a_). In contrast, in RBA only bound percent (B%) signals are reported, and only in selected studies affinity constants are estimated from displacement radioimmunoassays (RIA) [8].

Some type 1 DM-related autoantibodies have been previously measured by SPR. For example Ayela et al. [11] quantified autoantibodies to tyrosine phosphatase IA-2, and Carlsson et al. [12] described an indirect competitive immunoassay for detection and relative quantification of IAA. However there are no reports on both concentration and affinity assessment for IAA/PAA in patients' sera using the SPR technique.

The aim of this work was to use this novel technology to characterize the concentration and affinity of PAA present in sera from both childhood-onset and adult-onset diabetic patients with two alternative forms of proinsulin antigen: the genuine unmodified proinsulin (PI) and the recombinant chimeric thioredoxin-proinsulin (TrxPI) constructed in our laboratory [13]. The intention of this approach was to improve the orientation of PI assuring that all epitopes could be better exposed to antibodies. The final purpose was to further define the quantitative pattern of PAA response in rapid and slowly progressive forms of autoimmune diabetes otherwise well characterized in terms of clinical presentation, markers profile [14] and genetic susceptibility background [15].

## Methods

### Samples from diabetic patients

All samples from 51 selected diabetic patients that were included in this study spanned a wide range of PAA positivity.

Sera from 28 children and adolescents admitted to the Nutrition Service at the J. P. Garrahan Pediatric Hospital (Buenos Aires, Argentina), with a mean age of 8.31±4.20 at diagnosis were collected before or within 72 h of insulin treatment initiation. Type 1 diabetes was diagnosed according to WHO criteria [16]. The starting group included 71 children and adolescents attending the service from June 1994 to July 1996. As the hospital is a referral centre, patients came from all over Argentina and were mainly Caucasians. All these patients were tested in parallel for diabetic humoral markers, PAA, glutamic acid decarboxylase autoantibodies (GADA), protein tyrosine phosphatase IA-2 autoantibodies (IA-2A) and autoantibodies to zinc transporter 8 (ZnT8A). Most patients, 71.8%, were GADA+, the second marker in frequency was ZnT8A (69.0%), 66.2% were IA-2A+ and 36.6% were PAA+.

Sera from 23 elderly subjects attending the Diabetes Division at the José de San Martín Clinical Hospital (Buenos Aires, Argentina), with diabetes diagnosed at ≥65 years old and body mass index (BMI) <30 were included. These sera were selected from a starting population of 68 subjects attending the Diabetes Division from 2010 to 2011. All these adult-onset diabetic patients were tested in parallel for all the mentioned humoral markers. Out of these patients, 60.0% presented at least one humoral marker, 38.5% were GADA+, 1.5% were IA-2A+, 16.9% were ZnT8A+ and 32.3% were PAA+. Diagnosis was performed according to the American Diabetes Association [1]. The criteria for oral hipoglycaemic agent (OHA) therapy were those of the UK Prospective Diabetes Study [17]. Adult patients included in this study had not been treated with insulin before blood sample collection for immunochemical analysis.

Blood samples were collected after overnight fasting and sera were stored at −20°C until assayed. The collection of serum samples from newly diagnosed type 1 diabetic patients and adult-onset diabetic patients, and the respective protocols were approved by the Ethical Committees of the J. P. Garrahan National Pediatric Hospital and José de San Martín Clinical Hospital, respectively. Written consent from all participants involved in this study, and parental consent when being a minor, was obtained.

### Rabbit polyclonal antibodies to proinsulin

High-titered PI antibodies were obtained by immunizing two New Zealand White rabbits with 0.1 mg of standard human PI (Eli Lilly, Indianapolis, IN) emulsified in complete Freund's adjuvant. The initial injection was followed by booster injections with 0.1 mg of PI in incomplete adjuvant at three-week intervals. Rabbits were bled 15 days after the last boosting. They were maintained in a specific pathogen–free facility and treated with humane care in accordance with the Animal Care and Research Committee of the University of Buenos Aires.

### PAA detection by Radioligand Binding Assay (RBA)

Sera from diabetic patients were first subjected to RBA to detect the presence of PAA. Those positive sera were then used for SPR analysis in order to characterize their concentration and affinity parameters.

RBA for PAA was performed as previously described [18]. Briefly, cDNA coding for human PI was transcribed and translated using a rabbit reticulocyte lysate system in the presence of [^35^S]cysteine (New England Nuclear, Boston, MA), according to the manufacturer instructions. After overnight refolding, favored by a disulphide reduction-reoxidation procedure, [^35^S]-PI was isolated by reverse-phase HPLC. Sera (30 µl) were incubated for seven days at 4°C with 1,000 cpm of [^35^S]-PI in 90 µl of RBA buffer (50 mM sodium phosphate, 100 mM NaCl, pH 7, 0.1% Aprotinin and 0.1% bovine serum albumin). Subsequently, 50 µl of a 50% suspension of Protein G-Sepharose 4B FF (Amersham Biosciences, Piscataway, NJ) in RBA buffer were added, in order to isolate the immunocomplexes. Samples were centrifuged and supernatants were discarded. Pellets were washed four times with 200 µl of RBA buffer, suspended in 100 µl 1% SDS, and centrifuged (5 min at 6,000×g). Supernatants were carefully transferred to appropriate vials for scintillation counting, which was performed at 5 min per tube. Results were calculated as *B* % = 100×bound cpm/total cpm and expressed as *SD score* = (*B* %-*B*_C_ %)/*SD*_C_, where *B*_C_ % was the mean B % of control sera and *SD*_C_ its standard deviation. An assay was considered positive if *SD score* >3. Thirty normal controls were included and *B*_C_ % was normally distributed. The intra-assay CVs in triplicates were 10.34 and 3.78% for a SD score of 17.2 and 33.3, respectively.

### Fluid phase dose-response curves and data processing

The RIA carried out with the rabbit polyclonal serum against PI was performed by incubating 30 µl of 1/100 final dilution of this serum for seven days at 4°C with 1,000 cpm of [^35^S]-PI in the presence of 90 µl of different concentrations of human PI in RBA buffer. Immunocomplexes were isolated with Protein G-Sepharose 4B FF, the pellets were washed and suspended in 1% SDS. Radioactivity of supernatants was counted in an automatic beta counter. Concentration (q_0_) and median affinity constant (K_0_) parameters for the polyclonal rabbit anti-PI serum were derived from B/F = f (Log F or Log dose) plots [19].

### Recombinant Human Proinsulin

Standard PI was a gift from Eli Lilly (Indianapolis, IN). On the other hand, PI was expressed in *E. coli* as a fusion protein with thioredoxin (TrxPI) (Materials and Methods S1) as published elsewhere [13]. Briefly, *E. coli* GI 724 transformed with pTrx-PI were cultured at 30°C in 0.2% casein amino acids, 0.5% glucose, 1 mM MgCl_2_, and 100 µg/ml ampicillin. Protein expression was induced with 100 µg/ml tryptophan. Inclusion bodies were washed with 2 M urea in Tris 0.1 M pH 8.5 and solubilized with 5 M urea in Tris 0.1 M pH 8.5. Oxidative refolding was performed as described previously [18]. After overnight incubation for refolding of solubilized inclusion bodies, TrxPI was isolated by FPLC on a Q-Sepharose column (GE Healthcare, Sweden).

### Immobilization of PI or TrxPI for SPR assays

PI or TrxPI immobilization and subsequent interactions analysis were performed according to the user manual of the SPR BIAcore T100 biosensor (BIAcore, GE Healthcare, Uppsala, Sweden). Both proteins were immobilized on a carboxymethylated dextran sensor surface CM5 chip using conventional carbodiimide coupling chemistry [20]. The carboxyl groups at the sensor chips were activated with 0.2 M N-ethyl-N-(3-diethylmiopropyl) carbodiimide (EDC) and 0.05 M N-hydroxysuccinimide (NHS) for 7 min. PI and TrxPI diluted at 30 µg/ml in 10 mM acetate buffer (pH 4.5 and pH 4.0, respectively) were injected for surface immobilization until 2000 or 500 resonance units (RU) were obtained for concentration or affinity assay, respectively. The remaining reactive groups on the surfaces were blocked by injecting ethanolamine hydrochloride (1 M, pH 8.5, 7 min). The rabbit anti-human-PI serum was used as control of specific binding to immobilized PI or TrxPI.

To measure serum concentration of PAA, standard PI was immobilized on a CM5 sensor chip, being the immobilized protein in an amount corresponding to 1600 RU. For PAA affinity determination, the sensor chip was prepared by using lower concentrations of immobilized antigens (300 RU and 466 RU, for PI and TrxPI, respectively).

### SPR analysis

#### Concentration of serum proinsulin autoantibodies

A standard curve (RU vs. antibody concentration previously determined by RIA) was prepared by using the rabbit anti-human-PI polyclonal serum. The concentrations used were: 75 nM, 37.50 nM, 18.75 nM, 9.38 nM, 6.69 nM, 2.34 nM, 1.17 nM and 0.59 nM. On the other hand, sera from PAA positive patients were diluted ½, ¼ and **^1^/_8_** in running buffer (0.14 M NaCl, 2.7 mM KCl, 1.5 mM KPO_4_H_2_, 8.1 mM Na_2_PO_4_H, pH 7.4, 0.05% Tween 20), and samples were injected over 120 s. The binding rates were measured after 120 s in running buffer. After each antigen-antibody interaction, a regeneration step was carried out using 10 mM glycine-HCl, pH 1.5. All sensorgrams were corrected by subtracting the signal from the reference flow cell. For each patient the concentration of PAA was determined by comparison to the standard curve. All experiments were carried out at 25°C with a flow rate of 10 µl/min.

#### Analysis of serum proinsulin autoantibodies affinity

To evaluate the affinity of PAA, each patient's serum was used pure or diluted ½, ¼ and **^1^/_8_** in running buffer. In either case these starting samples were diluted ½ with CM dextran and NaCl to a final concentration of 1 mg/ml and 0.35 M, respectively, in order to eliminate the nonspecific reaction. Each sample was injected over 300 s and the binding rate in running buffer was measured after 300 s. Assays were carried out at 20°C using a flow rate of 10 µl/min. The association rate constant (k_1_), the dissociation rate constant (k_−1_), and the equilibrium constant (K_a_), were calculated from the sensorgrams analysis using the BIA-evaluation software.

#### Statistical analysis

Data are presented as median and range (min-max). Differences between groups were evaluated by the Mann-Whitney U-Test for comparison of the results derived from childhood-onset and adult-onset diabetic group samples. P values less than 0.05 were considered as significant. Correlation between K_a_ values obtained with immobilized PI or TrxPI was assessed by standard linear regression.

## Results

### Proinsulin antibodies parameters

#### Concentration of proinsulin autoantibodies in sera

**Figure 1:**
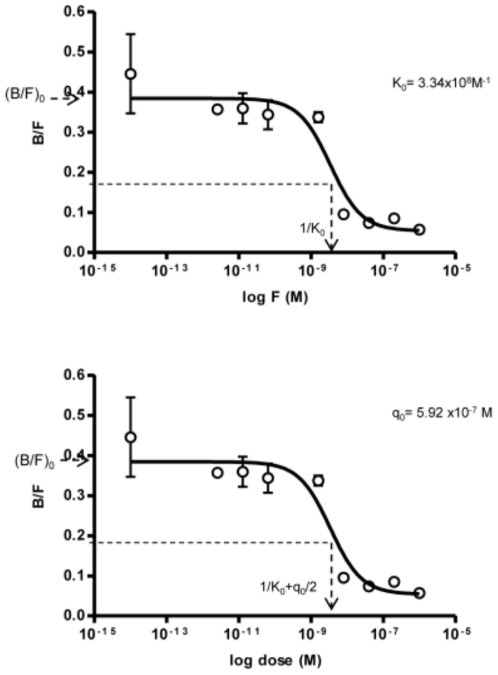
Dose-response curves obtained by using rabbit anti-human-PI serum and standard PI in a fluid phase displacement experiment incubated until reaching equilibrium. The parameter 1/K_0_ was calculated by interpolation at (B/F)_0_/2 in the upper graphic [B/F = f(log F)]. In the lower graphic representing B/F = f(log dose) the (B/F)_0_/2 intercepts the abscisa axis at 1/K_0_+q_0_/2 [21].

**Figure 2:**
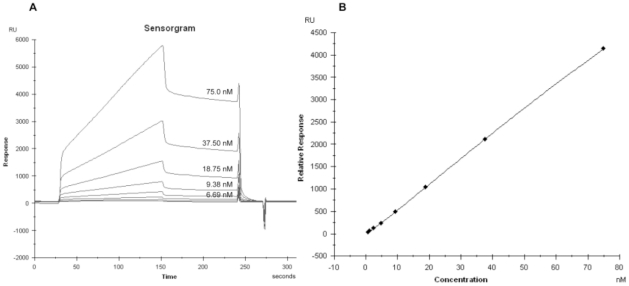
Calibration curve. (A) Sensorgram and (B) Standard curve of resonance units (RU) vs. antibody concentration as determined with an anti-human-PI polyclonal serum. The concentration values of polyclonal antibody used in the standard curve were: 75 nM, 37.50 nM, 18.75 nM, 9.38 nM, 6.69 nM, 2.34 nM, 1.17 nM and 0.59 nM.

The rabbit polyclonal anti-human-PI serum was first used to perform the calibration curve (Resonance Units –RU- vs. antibody concentration) which was later used to quantify PAA in patients' sera. The antibody concentration of the rabbit polyclonal serum was previously determined by RIA. Figure 1 shows the dose-response curves obtained in this fluid phase displacement experiment in which the incubation lasted until reaching equilibrium. The parameter K_0_ (3.34×10^8^ M^−1^) was calculated from the interpolation of 1/K_0_ at (B/F)_0_/2 in the plot B/F = f(log F). The parameter q_0_ (5.92×10^−7^ M) was calculated from the RIA plot B/F = f(log dose) where the (B/F)_0_/2 value intercepting the abscissa axis equals 1/K_0_+q_0_/2 [21]. The standard curve of SPR analysis was constructed by using the BIAcore™ software (Fig. 2).

**Figure 3:**
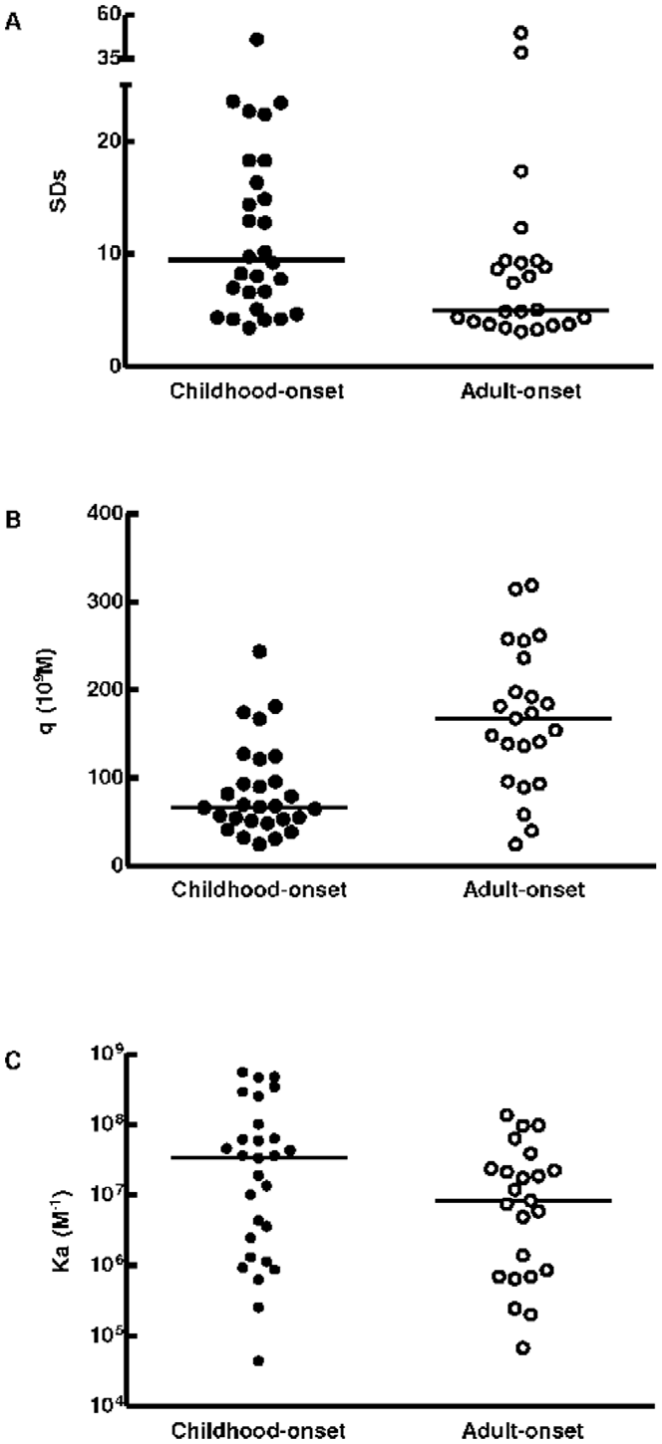
RBA and SPR results. (A) SD scores, (B) concentration (q) and (C) affinity constant (K_a_) obtained from 28 newly diagnosed type 1 diabetic patients and 23 adult-onset diabetic patients. All the parameters differed significantly between both groups of patients (p<0.05).

Sera from childhood-onset and adult-onset diabetic patients were first subjected to RBA to detect the presence of PAA. Those positive sera were then used for SPR analysis, in order to characterize PAA concentration (q) and affinity (K_a_) parameters (Fig. 3). In the 28 childhood-onset diabetic patients q ranged from 24.08×10^−9^ M to 243.65×10^−9^ M, median 67.12×10^−9^ M, and in the 23 adult-onset diabetic patients q ranged from 24.10×10^−9^ M to 318.4×10^−9^ M, median 167.40×10^−9^ M (Fig. 3b, Table 1 and Table S1). PAA were not detected in any of the normal human sera.

**Table 1:** Comparison of antigen-antibody interaction parameters: Standard Deviation Score (SDs) of signals obtained by RBA; antibody concentration (q) and affinity (K_a_) calculated by BIAevaluation software analysis (BIAcore™).

	SDs		q (10^−9^ M)		K_a-PI_ (10^6^ M^−1^)		K_a-TrxPI_ (10^6^ M^−1^)	
Patients	Median	Range (min-max)	Median	Range (min-max)	Median	Range (min-max)	Median	Range (min-max)
Childhood-onset	9.49	3.44–45.96	67.12	24.08–243.70	35.0	0.04–562.0	46.45	0.06–518.0
Adult-onset	5.04	3.10–49.60	167.4	24.10–318.40	8.35	0.07–136.0	7.16	0.05–183.0

The analyses were carried out on 28 childhood-onset and 23 adult-onset diabtetic patients.

All the parameters differed significantly between both groups of patients (p<0.05).

#### Affinity of proinsulin antibodies in rabbit and human sera

To measure the affinity of specific antibodies present in rabbit serum and patients' sera, a 1∶1 binding protocol was used for the fitting model. As controls, the median affinity of the rabbit polyclonal serum for PI and TrxPI was tested in parallel by a conventional fluid phase RIA (K_0-PI_ = 3.34×10^8^ M^−1^, K_0-TrxPI_ = 1.70×10^8^ M^−1^) and SPR analysis (K_a-PI_ = 2.48×10^8^ M^−1^, K_a-TrxPI_ = 2.01×10^8^ M^−1^). The affinity of PI antibodies measured by both procedures was in reasonable agreement, either using PI or TrxPI.

**Figure 4:**
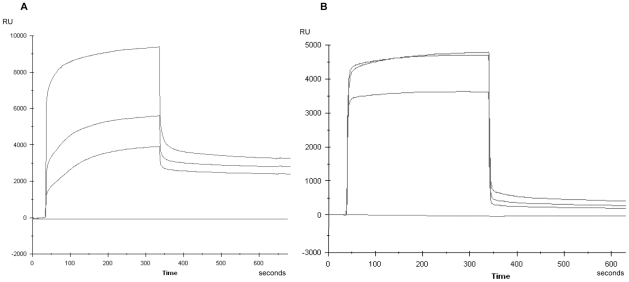
Representative sensorgrams. Panel (A): sera from a childhood-onset diabetic patient and panel (B): sera from an adult-onset diabetic patient.

Figure 4 shows representative examples of sensorgrams obtained for samples from a childhood-onset diabetic patient (a) and an adult-onset diabetic patient (b) at 3 serum dilutions.

In childhood-onset diabetic patients, the K_a-PI_ ranged from 4.45×10^4^ M^−1^ to 5.62×10^8^ M^−1^ and K_a-TrxPI_ 5.90×10^4^ M^−1^ to 5.18×10^8^ M^−1^. In patients with adult-onset diabetes, K_a-PI_ ranged from 6.78×10^4^ M^−1^ to 1.36×10^8^ M^−1^ and K_a-TrxPI_ ranged from 4.52×10^4^ M^−1^ to 1.83×10^8^ M^−1^. The median K_a-PI_ and K_a-TrxPI_ values were significantly higher in the childhood-onset diabetes group than in the adult-onset diabetes group (3.50×10^7^ M^−1^ vs 0.84×10^7^ M^−1^, 4.65×10^7^ M^−1^ vs 0.72×10^7^ M^−1^ respectively; p<0.05) (Fig. 3c, Table 1 and Table S1).

**Figure 5:**
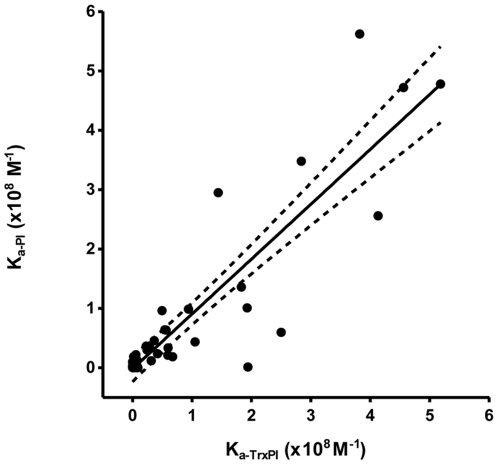
Correlation between K_a_ obtained using TrxPI or PI in SPR assays. K_a_ results were achieved by SPR using either TrxPI or PI as the immobilized antigen on sensor chip. The regression slope was 0.86±0.07 and the correlation coefficient (r^2^) was 0.80.

Besides, when injecting sera to flow cell immobilized with either PI or TrxPI, there was a satisfactory correlation between K_a_ values, with r^2^ = 0.80 (Fig. 5).

In order to evaluate the presence of antibodies to Trx in patients sera, a high sensitive chemiluminescence assay was performed (Supplemental Material and Methods S2). With this assay we have demonstrated that there were not IgG binding to Trx in the 51 patients sera studied (Supplemental Results S1 and Figure S1).

## Discussion

The first detectable sign of emerging beta cell autoimmunity during the preclinical asymptomatic period is the appearance of diabetes-related autoantibodies. For example, IAA have been shown to predict type 1 DM and to assist in the diagnosis of the disease [22], [23], [24]. IAA are detected in 43–70% of newly diagnosed type 1 diabetic patients [25], [26], but are most frequent and found at higher levels in young children [27], [28], [29].

There is no marker that allows to identify the IAA-positive children who will eventually become positive for multiple diabetes-related autoantibodies. Sustained or repeated antigen exposure results in a switch from IgM to IgG production, and subsequent exhaustion of antigen leads to the selection of clones that produce high-affinity antibodies to the antigen [30], [31]. Therefore, affinity could reflect a stage of antigen encounter and, in the case of IAAs, may be useful in staging the preclinical phase of type 1 DM. IAA affinity was previously measured in IAA-positive children from the prospectively followed BABYDIAB cohort [32]. The findings in these children who are followed during infancy indicate that IAA affinity is fixed relatively early in the autoimmune response, that it distinguishes IAAs with different epitope reactivity, and that it identifies IAA-positive children who will progress to multiple autoantibodies. Besides, Achenbach et al. [8] demonstrated that high affinity IAA were reactive to proinsulin, and Bömher et al. [33] found that PAA have a higher association with type 1 DM than IAA. However, we reasoned that concentration and affinity of autoantibodies define the level of such humoral response, so it may be important to determine both parameters for a better expression of the results. In this sense, the method based on the SPR technique described herein is an interesting approach for attaining this goal.

To calculate q and K_a_ of specific sera, the following sequence was performed: 1) sera from childhood-onset and adult-onset diabetic patients were subjected to reference RBA to detect the presence of PAA, and PAA positive sera were selected for the SPR analysis; 2) q of specific autoantibodies was determined by the use of a calibration curve; and 3) once q was known, K_a_ was determined by a kinetic procedure.

Because the immobilization of proteins on CM5 sensor chip is based on covalent binding between carboxyl groups of the chip surface and amino groups of the proteins, we decided to use as antigen a recombinant proinsulin fused to thioredoxin (TrxPI) produced in our laboratory. The goal of this approach was to improve the orientation of PI assuring that all epitopes were exposed to antibodies. In order to verify whether TrxPI is a useful tool for this assay, standard PI was also immobilized on another cell, and the results obtained with both proteins were compared. As shown in figure 5, there was a good correlation (r^2^: 0.80) between both immobilized antigens. Furthermore, some of the evaluated sera interact with higher affinity against TrxPI than against PI, reconfirming our assumption of an improved orientation of specific epitopes.

When analyzing comparatively samples from childhood-onset diabetic patients versus adult-onset diabetic patients the values obtained by conventional RBA were higher for the former group (SDs median 9.49 vs. 5.04). However, after SPR analysis, the childhood-onset diabetic patients presented lower PAA concentrations and higher affinities than the adult-onset diabetic group. These results are consistent with those obtained from solution RBA methods where the binding can be directly related to the intrinsic affinity of the antibody [21] (Fig. 3). On the other hand, the lower affinity values in adult-onset diabetic patients suggest a different etiopathogenic pathway associated to this slowly progressive form of autoimmune diabetes. In fact, a different autoantigen driving process must be involved in affinity maturation of IAA/PAA response in adult-onset diabetic patients since the lower affinity of their antibodies, as compared to those found in their type 1 diabetic counterparts, does not agree with their longer clinical course. All these data, altogether with the clinical differences, the dissimilar markers profile and the genetic background, suggest that there is a distinct autoimmune process involved in childhood-onset and adult-onset diabetic patients.

In conclusion, we have developed an immunoassay for the fast quantitation of PAA concentration and affinity in sera using the SPR technique. This technology has been shown to be useful for the characterization of PAAs parameters, q and K_a_, as an alternative to RIA, with high versatility and reproducibility associated to low occupational and environmental risk. In addition, the method offers a high analytical capacity in terms of sensitivity and time-saving.

Although this methodology is not eligible for routine marker screening, it may be advantageous in programs including longitudinal studies of first degree relatives of diabetic patients with demonstrable autoimmunity, specially when the conventional approach for determining positivity or negativity of humoral markers is not conclusive. Furthermore, a relevant contribution of this work is the application of the SPR technology to the indirect study of the etiopathogenesis of DM in patients with different clinical features, where the knowledge of autoantibodies parameters may allow to characterize more accurately the underlying autoimmune process against islet beta cells.

## Supporting Information

Materials and Methods S1 Expression vector of TrxPI.
(DOC)

Materials and Methods S2 Thioredoxin antibodies detection by Chemiluminescence Assay.
(DOC)

Results S1 Thioredoxin antibodies detection by Chemiluminescence Assay.
(DOC)

Figure S1 S.D. score (SDs) for the binding of Trx to control subjects (n = 40), positive control (polyclonal serum anti-Trx diluted 1/10000) and diabetic patients (n = 51) obtained by Chemiluminescence Assay. The cut-off value for the assay is indicated by a dotted line.
(TIF)

Table S1 Concentration (q) and affinity (K_a_) results obtained by SPR and RBA from 51 diabetic patients.
(DOC)
