# Supplementary material for: Surface Plasmon Resonance Reveals a Different Pattern of Proinsulin Autoantibodies Concentration and Affinity in Diabetic Patients
Source: PLoS One. 2012 Mar 19;7(3):e33574. doi: 10.1371/journal.pone.0033574 (PMC3307739; doi:10.1371/journal.pone.0033574)
Supplement: Materials and Methods S1 — Expression vector of TrxPI. (DOC) [file pone.0033574.s001.doc]

**Supplemental Materials and Methods S1**

Expression vector of TrxPI

pBR328, harbouring full‑length preproinsulin (PPI) cDNA, (kindly provided by Graeme Bell, University of Chicago, USA) was digested with *EcoRI*. The restriction fragment encoding PPI was cloned into pGem 3Zf (Promega, Madison, WI), yielding pGem3Zf‑PPI. The human PI gene was amplified by PCR from pGem3Zf‑PPI using 5' CCCAGCCATGGCCTTTGTGAACCAACACCTGT 3' and 5' TTTATTCGAGCTCTCTCGGTGCAGGAGGCG 3' primers. The amplification product incorporated *NcoI* and *SacI* sites at the 3’ and 5’ ends, respectively. The PCR product was ligated into the *SmaI* site of pGem3Zf to yield pGem3Zf‑PI. The identity of the new DNA molecule encoding for PI was corroborated by sequencing.

pGem3Zf‑PI was digested with *NcoI* and *EcoRI* and treated with S1 nuclease. The fragment containing the PI gene, with blunt 5' and 3' ends, was isolated and ligated into linearized pTrxFus (Invitrogen, Carlsbad, CA). The plasmid was linearized with *SmaI.* The resulting construct pTrx‑PI codes for the fusion protein thioredoxin-PI.
