# Supplementary material for: Surface Plasmon Resonance Reveals a Different Pattern of Proinsulin Autoantibodies Concentration and Affinity in Diabetic Patients
Source: PLoS One. 2012 Mar 19;7(3):e33574. doi: 10.1371/journal.pone.0033574 (PMC3307739; doi:10.1371/journal.pone.0033574)
Supplement: Materials and Methods S2 — Thioredoxin antibodies detection by Chemiluminescence Assay. (DOC) [file pone.0033574.s002.doc]

**Supplemental Materials and Methods S2**

Thioredoxin antibodies detection by Chemiluminescence Assay

*Reagents*. The coating buffer was PBS (0.14 mol/l NaCl, 2.7 mmol/l KCl, 1.5 mmol/l KPO4H2, 8.1 mmol/l Na2PO4H, pH 7.4), the blocking buffer was 3% skim milk in PBS and the dilution buffer was 3% skim milk in PBS/T (PBS containing 0.05% Tween 20). The chemiluminescent substrate was Luminigen® PPD (DPC®, Los Angeles, CA, USA)

diluted 1/10 in PBS. Alkaline Phosphatase -conjugated anti-rabbit / human IgG was from Jackson ImmunoResearch Laboratories, Inc. (West Grove, PA, USA).

Control sera used to establish the cut off value were from healthy subjects obtained from the Hemotherapy Division of the José de San Martín Clinical Hospital (Buenos Aires, Argentina). The collection of serum samples was approved by the Ethics Committee of the same hospital.

Antiserum against Trx, used as positive control, was obtained by immunizing a New Zealand White rabbit with 1 mg of recombinant Trx emulsified in complete Freund’s adjuvant. The initial injection was followed by booster injections with 1 mg of Trx in incomplete adjuvant at four-week intervals. The rabbit was bled 15 days after boosting.

Recombinant Trx was expressed in *E. coli* with the pTrx vector (Invitrogen, San Diego, CA, USA) and purified by osmotic shock according to the manufacturer’s instructions. The product was dialyzed against PBS and lyophilized.

*Chemiluminescence Assay Protocol*. All samples and blanks were assayed in duplicate. Except when otherwise indicated, incubations were at room temperature.

Opaque solid white high binding 96 well plate (Corning Costar Corporation, Cambridge, MA, Great Britain) were coated overnight at 4 °C with 0.5 μg of Trx, washed 5 times with PBS, blocked for 1.5 h with 300 μl of blocking buffer, and washed 5 times with PBS/T. Samples of 40 control human sera and of the 51 diabetic patients sera used in this work (50 μl/well) diluted 1/100 in dilution buffer containing non immunized rabbit serum (1/10000 final dilution) were incubated for 1 h. Positive control was diluted 1/10000 in dilution buffer. To measure the non-specific signal, duplicates of dilution buffer were incubated in wells in which serum had been omitted. After washing the plates 5 times with PBS/T, they were incubated for 1 h with Alkaline Phosphatase -conjugated anti-rabbit / human IgG (diluted 1:8000 in dilution buffer), followed by 5 washes with PBS/T. The chemiluminescent substrate (50 μl/well) was added and the plates were incubated in the dark for 10 min. The reaction was measured with 1420 Wallac Multilabel Counter VICTOR^3^™ (PerkinElmer Inc., USA).

The specific signal (SS) was calculated as the mean of the sample minus the mean of the non-specific control. Data were processed and results were expressed as SD score = (SS – SS_c_)/SD_c_, where SS_c_ is the mean SS of control sera and SD_c_ is its standard deviation. An assay was considered positive if SD score > 3.
