# Supplementary material for: Surface Plasmon Resonance Reveals a Different Pattern of Proinsulin Autoantibodies Concentration and Affinity in Diabetic Patients
Source: PLoS One. 2012 Mar 19;7(3):e33574. doi: 10.1371/journal.pone.0033574 (PMC3307739; doi:10.1371/journal.pone.0033574)
Supplement: Results S1 — Thioredoxin antibodies detection by Chemiluminescence Assay. (DOC) [file pone.0033574.s003.doc]

**Supplemental Results S1**

Thioredoxin antibodies detection by Chemiluminescence Assay

The assay developed was able to detect the rabbit polyclonal anti-Trx serum in a dilution 1/100000, demonstrating its high sensitivity. With this assay the 51 diabetic patients’ samples were analyzed and all of them resulted negative for antibodies to Trx.

These results are shown in Figure S1.
