# Supplementary figures and images for: Surface Plasmon Resonance Reveals a Different Pattern of Proinsulin Autoantibodies Concentration and Affinity in Diabetic Patients
Source: PLoS One. 2012 Mar 19;7(3):e33574. doi: 10.1371/journal.pone.0033574 (PMC3307739; doi:10.1371/journal.pone.0033574)

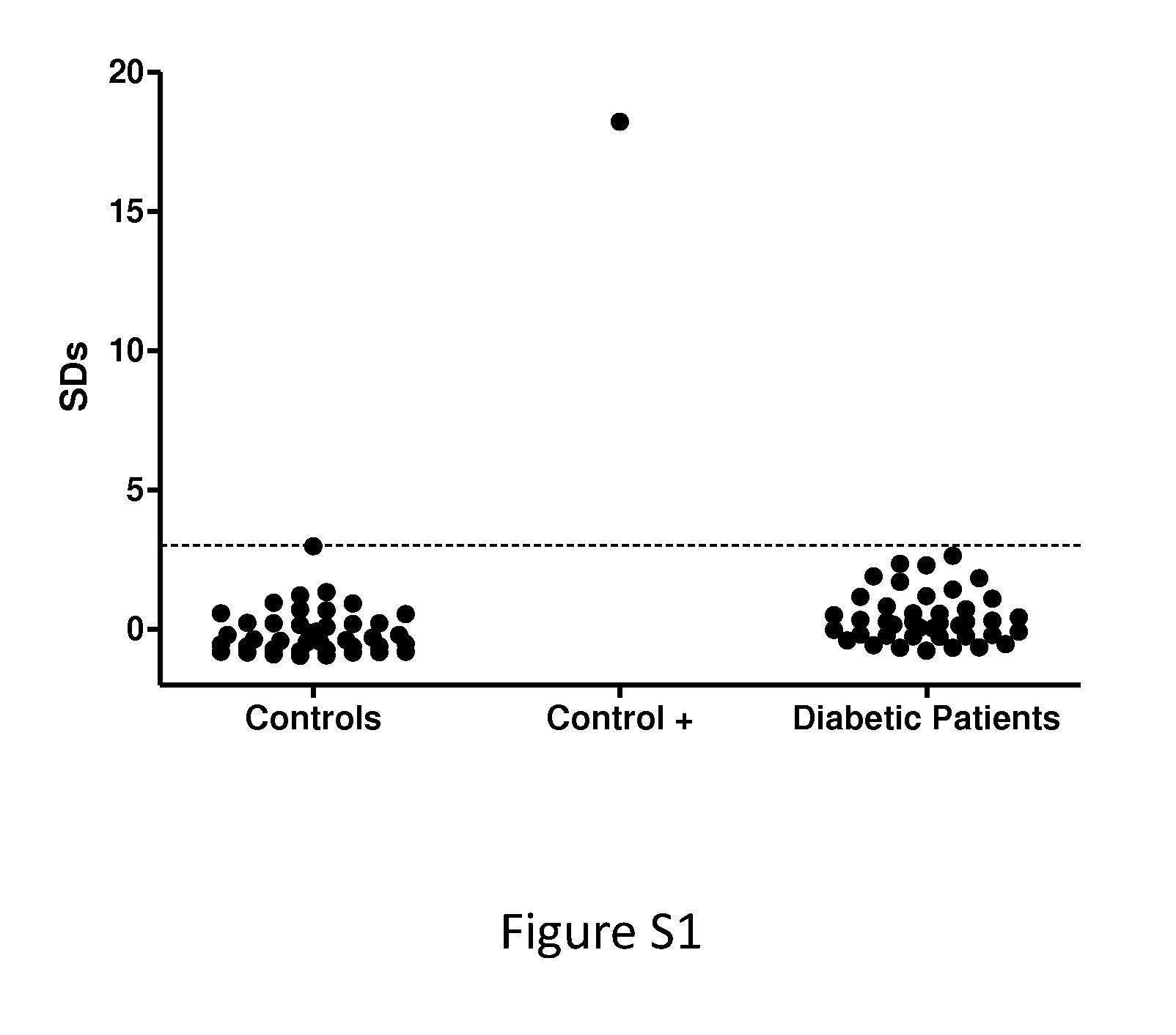

Supplement: Figure S1 — S.D. score (SDs) for the binding of Trx to control subjects (n = 40), positive control (polyclonal serum anti-Trx diluted 1/10000) and diabetic patients (n = 51) obtained by Chemiluminescence Assay. The cut-off value for the assay is indicated by a dotted line. (TIF) [file pone.0033574.s004.tif]
