# Supplementary material for: Surface Plasmon Resonance Reveals a Different Pattern of Proinsulin Autoantibodies Concentration and Affinity in Diabetic Patients
Source: PLoS One. 2012 Mar 19;7(3):e33574. doi: 10.1371/journal.pone.0033574 (PMC3307739; doi:10.1371/journal.pone.0033574)
Supplement: Table S1 — Concentration (q) and affinity (Ka) results obtained by SPR and RBA from 51 diabetic patients. (DOC) [file pone.0033574.s005.doc]

**Supplemental Table S1.** Concentration (q) and affinity (K_a_) obtained by SPR and RBA results from 51 diabetic patients

| **Diabetic Patients** | **q (x10^-9^M)** | **CV%** | **K_a hPI_** | **CV%** | **K_a TrxPI_** | **CV%** | **RBA** |
| --- | --- | --- | --- | --- | --- | --- | --- |
| **Childohood-onset** |  |  | **(x10^7^M^-1^)** |  | **(x10^7^M^-1^)** |  | **SDs** |
| 1 | 32.12 | 7.01 | 25.600 | 5.61 | 41.300 | 6.30 | 4.2 |
| 2 | 121 | 5.09 | 0.036 | 10.74 | 0.006 | 12.89 | 8.04 |
| 3 | 181.3 | 3.25 | 1.350 | 15.36 | 0.751 | 12.67 | 9.75 |
| 4 | 54.3 | 6.66 | 0.093 | 3.51 | 0.026 | 5.39 | 4.67 |
| 5 | 41.1 | 5.45 | 47.800 | 5.47 | 51.800 | 8.53 | 14.88 |
| 6 | 127 | 6.15 | 0.062 | 9.51 | 0.072 | 11.63 | 8.23 |
| 7 | 24.08 | 4.21 | 47.200 | 27.74 | 45.600 | 19.3 | 6.57 |
| 8 | 48.02 | 9.80 | 56.200 | 5.53 | 38.200 | 7.03 | 18.3 |
| 9 | 38.12 | 4.92 | 3.350 | 4.31 | 5.970 | 8.88 | 4.14 |
| 10 | 174 | 5.21 | 0.0875 | 28.79 | 0.020 | 2.70 | 14.39 |
| 11 | 243.65 | 15.65 | 0.004 | 4.03 | 0.002 | 5.74 | 45.96 |
| 12 | 89.92 | 14.33 | 29.500 | 10.86 | 14.400 | 2.59 | 18.3 |
| 13 | 92.74 | 7.15 | 6.210 | 17.30 | 6.614 | 15.18 | 6.69 |
| 14 | 65.67 | 11.74 | 1.890 | 27.58 | 6.730 | 18.44 | 22.67 |
| 15 | 53.25 | 5.09 | 34.800 | 16.07 | 28.400 | 48.89 | 7.78 |
| 16 | 95.38 | 4.22 | 4.370 | 5.56 | 10.500 | 15.57 | 4.22 |
| 17 | 69.14 | 23.42 | 5.970 | 7.54 | 25.000 | 3.61 | 23.42 |
| 18 | 64.39 | 10.17 | 0.114 | 4.68 | ND | - | 10.17 |
| 19 | 66.43 | 6.98 | 6.390 | 5.80 | 5.640 | 8.24 | 6.98 |
| 20 | 67.8 | 6.67 | 10.100 | 7.62 | 19.300 | 5.28 | 23.55 |
| 21 | 51.13 | 5.09 | 3.650 | 17.02 | 2.320 | 7.8 | 5.09 |
| 22 | 81.54 | 12.94 | 4.610 | 6.56 | 3.650 | 12.55 | 12.94 |
| 23 | 56.89 | 16.35 | 0.132 | 17.12 | 19.400 | 10.98 | 16.35 |
| 24 | 78.69 | 12.8 | 3.670 | 5.02 | 2.850 | 3.40 | 12.8 |
| 25 | 54.855 | 7.84 | 1.010 | 10.83 | 0.022 | 3.64 | 9.23 |
| 26 | 124.4 | 11.36 | 0.025 | 9.39 | 0.063 | 2.15 | 22.4 |
| 27 | 30.95 | 2.48 | 0.248 | 24.89 | 0.014 | 24.90 | 3.44 |
| 28 | 166.75 | 13.57 | 0.435 | 25.61 | 0.238 | 6.47 | 4.35 |
| **Adult-onset** |  |  |  |  |  |  |  |
| 1 | 197.5 | 6.82 | 6.400 | 4.71 | 5.450 | 3.46 | 3.1 |
| 2 | 255.4 | 3.28 | 0.835 | 8.57 | 0.227 | 16.81 | 38.4 |
| 3 | 236.1 | 3.38 | 2.150 | 7.62 | 5.940 | 7.82 | 49.6 |
| 4 | 257.35 | 16.24 | 2.380 | 35.62 | 4.200 | 20.54 | 3.65 |
| 5 | 93.11 | 3.73 | 13.600 | 5.48 | 18.300 | 7.28 | 4.35 |
| 6 | 147.8 | 5.90 | 1.210 | 5.43 | 3.140 | 4.72 | 5.04 |
| 7 | 173.5 | 2.98 | 0.070 | 8.91 | 0.893 | 9.65 | 4,00 |
| 8 | 184.3 | 3.38 | 2.239 | 8.72 | 0.578 | 10.28 | 17.36 |
| 9 | 136.15 | 3.76 | 0.020 | 6.78 | 0.288 | 10.02 | 12.33 |
| 10 | 192.15 | 3.17 | 0.086 | 3.52 | 0.336 | 2.54 | 3.48 |
| 11 | 167.4 | 15.04 | 0.025 | 7.47 | 0.775 | 7.51 | 8.86 |
| 12 | 181.4 | 8.24 | 3.938 | 19.37 | 2.446 | 8.87 | 4.35 |
| 13 | 95.34 | 8.81 | 0.018 | 11.74 | 0.015 | 17.48 | 3.3 |
| 14 | 89.05 | 2.09 | 0.068 | 12.26 | 0.755 | 9.56 | 4.87 |
| 15 | 39.61 | 6.03 | 9.901 | 10.92 | 9.407 | 6.48 | 9.38 |
| 16 | 138 | 15.22 | 1.881 | 11.84 | 0.201 | 6.75 | 7.47 |
| 17 | 314.3 | 2.69 | 0.064 | 6.91 | 0.032 | 29.03 | 4.87 |
| 18 | 58.13 | 3.31 | 0.139 | 4.05 | 0.147 | 8.73 | 8.68 |
| 19 | 24.1 | 15.96 | 0.964 | 10.94 | 4.960 | 21.51 | 9.2 |
| 20 | 153.8 | 9.8 | 0.743 | 25.40 | 0.297 | 31.82 | 9.38 |
| 21 | 140.8 | 6.69 | 1.760 | 5.78 | ND | - | 8.03 |
| 22 | 318.4 | 2.32 | 0.592 | 4.65 | 0.582 | 11.30 | 3.76 |
| 23 | 261.5 | 3.48 | 0.493 | 8.64 | 0.678 | 3.48 | 3.77 |
